# Regulatory Roles of Noncanonical Inflammasomes in Inflammatory Lung Diseases

**DOI:** 10.3390/ijms26010027

**Published:** 2024-12-24

**Authors:** Young-Su Yi

**Affiliations:** Department of Life Sciences, Kyonggi University, Suwon 16227, Republic of Korea; ysyi@kgu.ac.kr; Tel.: +82-31-249-9644

**Keywords:** caspase-11, caspase-4, noncanonical inflammasome, inflammation, lung disease

## Abstract

The inflammatory response consists of two stages: priming and triggering. The triggering stage is marked by the activation of inflammasomes, which are cytosolic protein complexes acting as platforms for inflammation. Inflammasomes are divided into canonical and noncanonical categories. Inflammatory lung diseases such as asthma, chronic obstructive pulmonary disease (COPD), acute respiratory distress syndrome (ARDS), inflammatory lung injury, and pulmonary fibrosis arise from lung inflammation and damage. While the role of canonical inflammasomes in these diseases is well demonstrated, recent findings emphasize the critical roles of noncanonical inflammasomes in regulating inflammation and various inflammatory conditions. Particularly, new studies highlight their involvement in inflammatory lung diseases. This review delves into recent research on the regulatory roles of noncanonical inflammasomes, such as human caspase-4 and murine caspase-11, in lung inflammation and the development of inflammatory lung diseases, as well as the potential for targeting these inflammasomes for new treatments.

## 1. Introduction

Inflammation is a part of innate immunity characterized by two sequential phases: priming and triggering [1,2,3]. During the priming phase, inflammatory responses are prepared by the transcriptional activation of inflammatory molecules, while the triggering phase involves the activation of inflammasomes and cytosolic multiprotein complexes that initiate inflammatory responses [1,2,4,5]. Canonical inflammasomes, discovered earlier, include NLR inflammasomes such as NLRP1, NLRP3, NLRC4, NLRP6, NLRP9, and NLRP12, as well as non-NLR inflammasomes such as absence of melanoma 2 (AIM2), interferon γ-inducible protein 16 (IFI16), and pyrin inflammasomes, whereas more recently identified noncanonical inflammasomes include murine caspase-11, human caspase-4, and caspase-5 inflammasomes [5,6,7,8]. Inflammasomes are activated by recognizing a variety of pathogen-associated molecular patterns (PAMPs) and damage-associated molecular patterns (DAMPs) through pattern recognition receptors (PRRs). Despite differences in activation, canonical and noncanonical inflammasomes share common downstream inflammatory signaling pathways [5,6,7,8,9]. Activation of inflammasomes leads to the proteolytic cleavage of gasdermin D (GSDMD), with the resulting N-terminal fragments (N-GSDMD) forming pores in cell membranes, causing an inflammatory cell death known as pyroptosis [5,6,7,8,9]. Simultaneously, inflammasome activation also results in the proteolytic cleavage of caspase-1, which in turn leads to the maturation and release of pro-inflammatory cytokines like IL-1β and IL-18 through the GSDMD pores [5,6,7,8,9]. Numerous studies have highlighted the critical role of canonical inflammasomes, particularly the NLRP3 inflammasome, in driving inflammatory responses and various human diseases [6,9,10,11,12]. Recently, growing evidence has pointed to noncanonical inflammasomes as novel and important players in inflammatory responses and immunopathological conditions [13,14,15,16,17,18,19,20,21].

Pulmonary inflammation is triggered by exposure to airborne toxins, irritants, and respiratory infections, leading to the development of inflammatory lung diseases such as asthma, chronic obstructive pulmonary disease (COPD), acute respiratory distress syndrome (ARDS), inflammatory lung injury, and pulmonary fibrosis [22]. Inflammatory lung diseases are pathological conditions characterized by persistent inflammation in the pulmonary system, which leads to tissue damage and impaired pulmonary functions [23,24,25]. Inflammatory lung diseases are some of the most widespread diseases worldwide, and in the last decades, the number of patients with inflammatory lung diseases has grown, highlighting the need for medical treatment [26]. Studies have found that the activation of canonical inflammasomes, particularly the NLRP3 inflammasome, induces pulmonary inflammation and plays critical roles in various inflammatory lung diseases [27,28]. Notably, recent accumulating evidence suggests that noncanonical inflammasomes also play crucial roles in triggering pulmonary inflammation, contributing to the onset of various inflammatory lung diseases. This review discusses research on the regulatory roles of noncanonical inflammasomes in the development of inflammatory lung diseases and further emphasizes the potential of modulating their functions as a potential strategy for preventing and treating these diseases.

## 2. Noncanonical Inflammasomes

### 2.1. Classification and Molecular Structures

**Figure 1:**
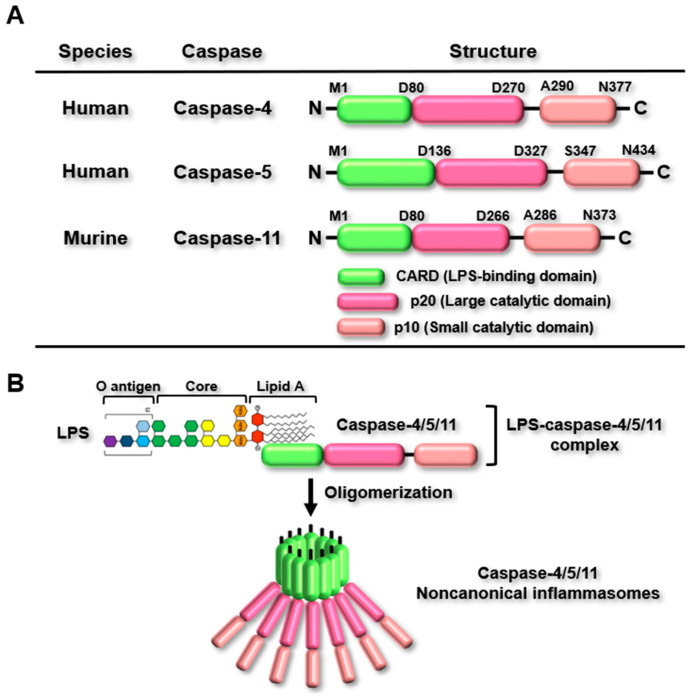
Classification and molecular structures of noncanonical inflammasomes. (**A**) Structure of human caspase-4/5 and murine caspase-11. Human caspase-4/5 and murine caspase-11 are composed of three domains: an N-terminal CARD (LPS binding domain), a p20 domain (large catalytic domain), and a C-terminal p10 domain (small catalytic domain). Their respective lengths are 377 amino acids for human caspase-4, 434 amino acids for human caspase-5, and 373 amino acids for murine caspase-11. (**B**) LPS detection by caspase-4/5/11. Caspase-4/5/11 directly interacts with the lipid A of LPS through their CARD domains, resulting in the formation of LPS-caspase-4/5/11 complexes. These complexes subsequently oligomerize to generate caspase-4/5/11 noncanonical inflammasomes.

As described earlier, there are two types of inflammasomes, and the molecular structures of canonical and noncanonical inflammasomes significantly differ. When the PRRs of canonical inflammasomes detect their specific PAMPs and DAMPs, they interact with caspase-1, either with or without the assistance of ASC, a bipartite adaptor, resulting in the assembly of canonical inflammasomes [5,6]. However, the PRRs of noncanonical inflammasomes, such as murine caspase-11 and human caspase-4 and caspase-5, detect their DAMPs without the involvement of both caspase-1 and ASC [7,8]. Murine caspase-11 and human caspase-4/5 share a similar molecular structure with identical domains. They all contain a caspase recruitment domain (CARD) at the N-terminus, a large catalytic domain (p20), and a small catalytic domain (p10) at the C-terminus (Figure 1A). Despite having the same domain structure, they vary in amino acid length, with caspase-4/5/11 containing 377, 434, and 373 amino acids, respectively (Figure 1A). Canonical inflammasomes can detect various types of PAMPs and DAMPs, but lipopolysaccharide (LPS), an endotoxin from Gram-negative bacteria, is recognized as the only PAMP detected by caspase-4/5/11 [7,8]. Caspase-4/5/11 recognizes LPS through a direct interaction between the CARD of caspase-4/5/11 and lipid A of LPS, which results in oligomerization via CARD-CARD interactions to generate caspase-4/5/11 noncanonical inflammasomes (Figure 1B). The oligomerized caspase-4/5/11 noncanonical inflammasomes are activated through autoproteolytic processing, which triggers noncanonical inflammasome-activated inflammatory signaling pathways.

### 2.2. LPS Internalization and Detection of LPS by Noncanonical Inflammasomes

LPS is an endotoxin found in the cell walls of Gram-negative bacteria, and following bacterial infection, LPS is internalized into host cells through various mechanisms. LPS binds to toll-like receptor 4 (TLR4) with the assistance of LPS-binding protein (LBP) and MD2, and the LPS-MD2-TLR4 complex is taken up by host cells via receptor-mediated endocytosis [1]. LPS also binds to another type of receptor, the receptor for advanced glycation end-product (RAGE), with the assistance of hepatocyte-related high-mobility group box 1 (HMGB1), and the LPS-HMGB1-RAGE complex enters the host cells via receptor-mediated endocytosis [1]. Gram-negative bacteria generate outer membrane vesicles (OMVs) that contain LPS, and these OMVs can merge with host cell membranes, leading to their internalization through receptor-mediated endocytosis [1].

For detection of LPS by caspase-4/5/11, the PRRs of noncanonical inflammasomes, endocytosed LPS in endosomes, and Gram-negative bacteria residing in cytosolic vacuoles must be released into the cytosol. Guanylate-binding proteins (GBPs), which are members of the interferon (IFN)-inducible GTPase family, attach to endosomes and vacuoles, causing membrane disruption, and this breakdown of membrane integrity allows LPS to access the cytosol, where it can interact with caspase-4/5/11 [1]. Caspase-4/5/11 recognizes cytosolic LPS through direct binding between the CARD domain of caspase-4/5/11 and the lipid A portion of LPS, resulting in the formation of LPS-caspase-4/5/11 complexes [7,8]. The LPS-caspase-4/5/11 complexes then undergo oligomerization through CARD-CARD interactions, leading to the assembly of noncanonical inflammasomes and subsequent activation of caspase-4/5/11 through autoproteolysis [7,8,29,30]. In murine caspase-11, autoproteolysis occurs at aspartic acid residue 285, with cysteine residue 254 serving as the active catalytic site for this processing [29]. However, the autoproteolysis of human caspase-4/5 and the molecular mechanisms behind it remain largely unknown.

### 2.3. Noncanonical Inflammasome-Activated Inflammatory Signaling Pathways

**Figure 2:**
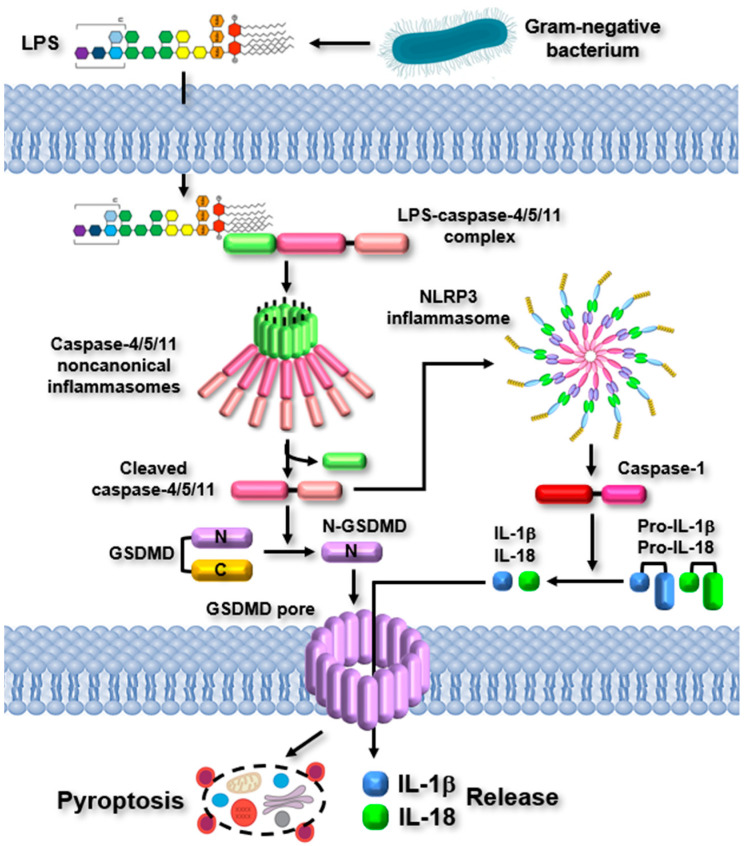
Noncanonical inflammasome-activated inflammatory signaling pathways. Inflammatory signaling pathways are activated by noncanonical inflammasomes. LPS from Gram-negative bacteria enters host cells and directly interacts with caspase-4/5/11, triggering the formation of caspase-4/5/11 noncanonical inflammasomes. These inflammasomes are activated through autoproteolytic cleavage, resulting in GSDMD pore-driven pyroptosis and the activation of the NLRP3 canonical inflammasome, which, in turn, leads to the proteolytic maturation and release of pro-inflammatory cytokines through GSDMD pores.

Upon the detection of LPS by caspase-4/5/11, the caspase-4/5/11 noncanonical inflammasomes are activated, initiating inflammatory responses. The activation of noncanonical inflammasomes triggers two major inflammatory signaling pathways. The activated noncanonical inflammasomes promote the proteolytic cleavage of GSDMD at the 276 aspartic acid residue, resulting in the formation of N-terminal and C-terminal fragments of GSDMD [7,8]. The N-terminal fragments of GSDMD (N-GSDMD) subsequently translocate to the cell membranes and oligomerize to form GSDMD pores, resulting in pyroptosis characterized by rapid membrane rupture and the release of inflammatory molecules [7,8]. Simultaneously, the activated noncanonical inflammasomes stimulate the NLRP3 canonical inflammasome by promoting potassium ion (K+) efflux, a crucial event necessary for NLRP3 inflammasome activation, via GSDMD pores, the P2X7 channel, bacterial pore-forming toxins, and membrane damage [1]. The NLRP3 inflammasome, activated by the noncanonical inflammasome, promotes the proteolytic activation of caspase-1. This results in the caspase-1-mediated maturation and release of pro-inflammatory cytokines, IL-1β and IL-18, as well as various inflammatory molecules through GSDMD pores [7,8]. The released pro-inflammatory cytokines and inflammatory molecules enhance inflammatory responses by activating other types of immune cells. The noncanonical inflammasome-activated inflammatory signaling pathways are described in Figure 2.

## 3. Regulatory Roles of Noncanonical Inflammasomes in Inflammatory Lung Diseases

### 3.1. Asthma

Asthma is a chronic inflammatory disease that affects the airways of the lungs and impacts more than 300 million people globally, and another 100 million are expected to be at risk [31]. Asthma is characterized by narrow, swollen airways, increased mucus production, reversible airflow obstruction, and bronchospasms. Symptoms include difficulty breathing, persistent coughing, chest tightness, a whistling sound, and shortness of breath.

Recent studies have demonstrated the regulatory roles of the murine caspase-11 noncanonical inflammasome in the pathogenesis of asthma. Khweek et al. investigated the role of caspase-11 noncanonical inflammasome in regulating the host response to house dust mites (HDM) and resulting allergic asthma [32]. HDM and allergy-associated cytokines increased the expression of caspase-11 in mouse BMDMs; however, the production of pro-inflammatory cytokines decreased in caspase-11^−/−^ BMDMs in response to HDM [32]. Additionally, the total cellular infiltration into the bronchial alveolar lavage fluids (BALF) and the levels of pro-inflammatory cytokines in the BALF were significantly decreased in caspase-11^−/−^ mice in response to HDM [32]. Moreover, the histological signs of lung inflammation were reduced in caspase-11^−/−^ mice in response to HDM [32]. These findings indicate that murine caspase-11 noncanonical inflammasome exacerbates airway inflammation in response to HDM exposure and may play a role in the progression of HDM-induced asthma.

As previously described, human caspase-4 is homologous to murine caspase-11, and a study has identified the regulatory role of the human caspase-4 noncanonical inflammasome in allergic airway inflammation in asthma patients. Simpson et al. highlighted the involvement of the caspase-4 noncanonical inflammasome in patients with neutrophilic asthma, a form of asthma distinguished by elevated levels of neutrophils in the lungs and airways [33]. Neutrophilic asthma patients showed a marked increase in caspase-4 expression and elevated production of IL-1β and IL-18 [33]. Notably, the elevated expression and production of caspase-4, IL-1β, and IL-18 were not observed in other asthma types, such as eosinophilic and paucigranulocytic asthma [33], which strongly suggests that the caspase-4 noncanonical inflammasome plays a pivotal role in neutrophilic asthma rather than in eosinophilic or paucigranulocytic asthma. The differences in molecular mechanisms related to noncanonical inflammasome activation across different types of asthma require further investigation. Additionally, since macrophages play a crucial role in inflammasome activation, further research will be needed to investigate the role of the caspase-4 noncanonical inflammasome in macrophages from asthma patients with various asthma types.

Zasłona et al. explored the aggravating impact of the murine caspase-11 and human caspase-4 (caspase-4/11) noncanonical inflammasomes in allergic airway inflammation [34]. Caspase-11 expression increased in the airways of mice with OVA-induced allergic inflammation, and caspase-11 deficiency provided protection against allergic lung inflammation in these mice [34]. Prostaglandin E_2_ (PGE_2_) suppressed caspase-4/11 expression in mouse BMDMs, human monocyte-derived macrophages, and the airways of mice with OVA-induced allergic inflammation, resulting in the inhibition of pyroptosis driven by the activation of caspase-4/11 noncanonical inflammasomes [34]. These findings strongly indicate that caspase-4/11 noncanonical inflammasomes play a key role in allergic airway inflammation and may influence the pathophysiology of asthma. Agents, such as PGE_2_, which effectively inhibit caspase-4/11 noncanonical inflammasomes, could serve as potential treatments for asthma.

Cai et al. also reported the aggravating role of Dectin-1-activated caspase-4/11 noncanonical inflammasomes in neutrophil inflammation and asthma [35]. Dectin-1 activated the noncanonical caspase-11 inflammasome and triggered pyroptosis, leading to pulmonary neutrophil inflammation in house dust mite (HDM)-induced asthmatic mice and MH-S alveolar macrophages [35]. However, inhibition of caspase-11 noncanonical inflammasome alleviated Dectin-1-activated airway inflammation, the proteolytic activation of GSDMD, and pyroptosis, leading to reduced neutrophil inflammation in HDM-induced asthmatic mice and MH-S alveolar macrophages [35]. Moreover, Dectin-1 expression in macrophages showed a positive correlation with neutrophil inflammation and caspase-4 expression in asthma patients [35]. These results suggest the functional interplay between Dectin-1 and caspase-4/11 noncanonical inflammasomes to exacerbate the airway neutrophil inflammation and asthma, which indicates that caspase-4/11 noncanonical inflammasomes and Dectin-1 are potential targets for treating asthma.

**Figure 3:**
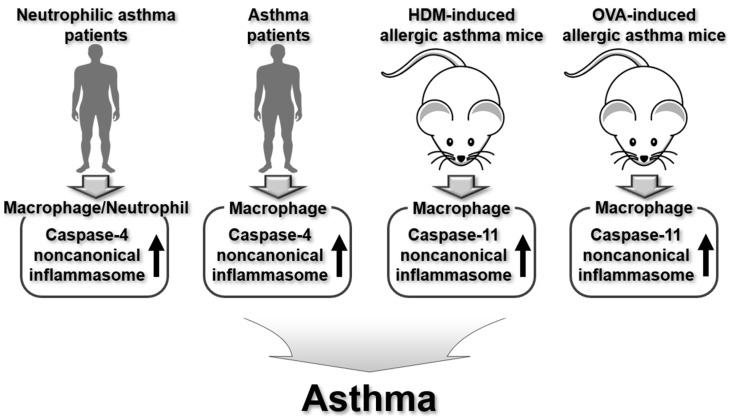
Regulatory roles of human caspase-4 and murine caspase-11 noncanonical inflammasomes in human asthma patients and animal models of allergic asthma.

In summary, caspase-4/11 noncanonical inflammasomes contribute to the worsening of airway inflammation and the development of asthma, as shown in animal models and human asthma patients. The regulatory roles of caspase-4/11 noncanonical inflammasomes in asthma pathogenesis are summarized in Figure 3.

### 3.2. Chronic Obstructive Pulmonary Disease (COPD)

Chronic Obstructive Pulmonary Disease (COPD) is a type of progressive lung disease characterized by chronic respiratory symptoms and airflow limitation due to abnormalities of the airways (bronchitis) and/or alveoli (emphysema) that cause persistent, often progressive, airflow obstruction [36]. COPD is the third leading cause of death globally, responsible for 3.5 million deaths annually, which accounts for approximately 5% of all deaths worldwide [36]. Smoking and air pollution are the most common causes of COPD, and the main symptoms of COPD include shortness of breath and a cough. While there is no effective therapy for COPD, it is preventable, and the symptoms can improve if patients avoid smoking and exposure to air pollution.

Research has shown that the murine caspase-11 noncanonical inflammasome plays a regulatory role in the development of COPD. Eltom et al. demonstrated the involvement of the caspase-11 noncanonical inflammasome in cigarette smoke (CS)-induced airway inflammation and the development of COPD [37]. The absence of the caspase-11 noncanonical inflammasome significantly reduced pro-inflammatory cytokine levels, neutrophilia, and airway inflammation in the BALF and lung tissues of CS-exposed COPD mice [37], indicating that the caspase-11 noncanonical inflammasome exacerbates CS-induced airway inflammation and contributes to COPD development.

Colarusso et al. also highlighted the regulatory role of the caspase-11 noncanonical inflammasome in CS-induced COPD. Exposure to CS led to alveolar enlargement, collagen deposition, and an increase in mucus and pro-inflammatory cytokine production in mouse lung tissues [38]. Notably, caspase-11 noncanonical inflammasome was activated in the lung tissues of CS-exposed mice, however, the CS-induced alveolar enlargement, collagen deposition, and the increase in mucus and pro-inflammatory cytokine production were reduced in the lung tissues of 129Sv mice, which have nonfunctional caspase-11 [38]. These findings indicate that the caspase-11 noncanonical inflammasome plays a key role in the lung inflammation observed in smoking-related COPD patients. However, the role of human caspase-4 noncanonical inflammasome should be further evaluated in clinical studies using COPD patients.

The same group further demonstrated the regulatory role of the human caspase-4 noncanonical inflammasome, in cooperation with the AIM2 canonical inflammasome, in the peripheral blood mononuclear cells (PBMCs) from exacerbated COPD patients [39]. The activation of the AIM2 inflammasome triggered the release of pro-inflammatory cytokines, such as interleukin (IL)-1α and tumor growth factor (TGF)-β in exacerbated PBMCs from COPD patients in a caspase-4 noncanonical inflammasome-dependent manner [39]. This suggests functional cooperation between caspase-4 noncanonical and AIM2 canonical inflammasomes in airway inflammation in COPD-derived exacerbated PBMCs. However, the molecular mechanisms underlying the functional cooperation of these two inflammasomes remain unclear and require further investigation.

*Moraxella catarrhalis* is a Gram-negative bacterium that can cause infection of the human respiratory system and is a leading cause of COPD exacerbation [40]. Tuipulotu et al. investigated the role of caspase-4/11 noncanonical inflammasomes in host responses to *M. catarrhalis* infection [41]. *M. catarrhalis* infection activated caspase-4/11 noncanonical inflammasomes, resulting in GSDMD-dependent pyroptosis and NLRP3 canonical inflammasome activation in mouse BMDMs and THP-1 human macrophages [41]. These results indicate that caspase-4/11 noncanonical inflammasomes play a crucial role in macrophage-driven immune responses to *M. catarrhalis* infection and COPD exacerbation triggered by *M. catarrhalis* infection. However, the role of caspase-4/11 noncanonical inflammasomes and the mechanisms involved in COPD exacerbation due to *Moraxella catarrhalis* infection need to be clarified in COPD animal models and human patients.

**Figure 4:**
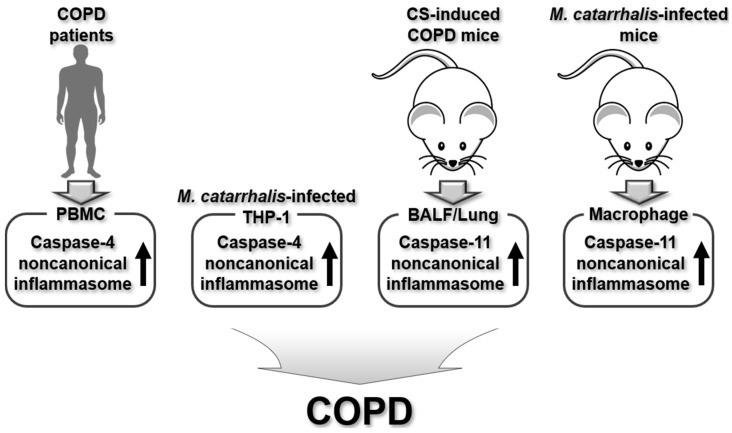
Regulatory roles of human caspase-4 and murine caspase-11 noncanonical inflammasomes in human COPD patients, human macrophages, and animal models of COPD.

In summary, caspase-4/11 noncanonical inflammasomes play a crucial role in COPD pathogenesis triggered by CS and Gram-negative bacterial infection, as well as in exacerbated PBMCs from COPD patients. The regulatory roles of caspase-4/11 noncanonical inflammasomes in COPD pathogenesis are summarized in Figure 4.

### 3.3. Acute Lung Injury (ALI) and Acute Respiratory Distress Syndrome (ARDS)

Acute lung injury (ALI) and the more severe acute respiratory distress syndrome (ARDS) are pulmonary and respiratory manifestations resulting from acute inflammation characterized by the breakdown of lung endothelial and epithelial barriers, pulmonary infiltrates, hypoxemia, and edema [42]. ALI and ARDS can be triggered by multiple factors, including sepsis, pathogen infections, inhalation of toxic substances, and thoracic trauma [42]. In recent decades, the incidence of ALI and ARDS has risen due to the coronavirus pandemic, as coronavirus infection causes severe pulmonary inflammation, leading to irreversible damage in the lung tissues, which is a hallmark of ALI and ARDS [43]. Despite the severity of ALI and ARDS, treatment options remain limited, with available therapies primarily including antibiotics and antiviral agents.

The role of noncanonical inflammasomes in the pathogenesis of ALI and ARDS has been demonstrated in animal models of these conditions, with ALI serving as the animal model that corresponds to human ARDS. Several animal models of ALI, such as cecal ligation and puncture (CLP) and LPS-induced ALI, have been developed for research purposes [44], and studies using CLP-induced ALI in mice have shown the involvement of the caspase-11 noncanonical inflammasome in the pathogenesis of ALI and ARDS. Xie et al. explored the functional collaboration of caspase-11 noncanonical inflammasome and High-mobility group box 1 (HMGB1) in ALI using a CLP-induced ALI mouse model [45]. HMGB1 aggravated lung injury and triggered severe inflammation in mice with CLP-induced ALI [45]. In ALI mice, levels of HMGB1 and caspase-11 increased in the lungs, while inhibiting HMGB1 reduced caspase-11 expression and pyroptosis in lung tissues [45]. These findings suggest that HMGB1 aggravates caspase-11 noncanonical inflammasome-dependent pyroptosis in ALI. However, the exact mechanism by which HMGB1 regulates the caspase-11 noncanonical inflammasome still remains to be elucidated.

Ding et al. also investigated the role of the HMGB1-caspase-11 axis in CLP-induced ALI mice [46]. Lung injury and inflammation were diminished in ALI mice lacking HMGB1 and its receptor, the receptor for advanced glycation end-products (RAGE) [46]. Moreover, lung injury and inflammation were also diminished in ALI mice lacking caspase-11 [46]. These results are consistent with a previous study by Xie et al. [45], which demonstrated that HMGB1 and the caspase-11 noncanonical inflammasome cooperate to aggravate ALI. However, the mechanism behind this cooperation remains unknown and to be elucidated.

Zhang et al. demonstrated the protective effect of luteolin by targeting the caspase-11 noncanonical inflammasome in CLP-induced ALI mice [47]. Luteolin, a natural flavonoid, reduced inflammation and lung injury in mice with CLP-induced ALI [47]. Additionally, the levels of caspase-11 and its downstream inflammatory molecules, including GSDMD and pro-inflammatory cytokines, were reduced in the lungs of ALI mice [47]. These observations indicate that the activation of the caspase-11 noncanonical inflammasome triggers pyroptosis and inflammation in ALI and that inhibiting the caspase-11 noncanonical inflammasome can improve ALI by reducing caspase-11-dependent pyroptosis and inflammatory responses.

As previously mentioned, LPS-induced ALI serves as another animal model for studying ALI and ARDS, and studies have highlighted the regulatory role of the caspase-11 noncanonical inflammasome in the development of ALI and ARDS using mice subjected to LPS-induced ALI. Endo et al. investigated the role of caspase-11 noncanonical inflammasome associated with the ER stress-C/EBP homologous protein (CHOP) pathway in the pathogenesis of lung inflammation and injury using LPS-induced ALI mice [48]. The caspase-11 noncanonical inflammasome was activated, triggering inflammatory responses in the lungs of LPS-induced ALI mice and primary peritoneal macrophages [48]. In LPS-induced ALI mice lacking CHOP, the activation of the caspase-11 noncanonical inflammasome was suppressed; however, it was activated in response to an ER stress inducer [48]. These findings indicate that the ER stress-CHOP pathway activates the caspase-11 noncanonical inflammasome, contributing to lung inflammation and the development of ALI.

Hu et al. investigated the role of caspase-11 noncanonical inflammasome associated with Basic helix-loop-helix family member e40 (Bhlhe40), a member of transcription factor subfamilies, in LPS-induced ALI mice [49]. Bhlhe40 was significantly expressed in the lung tissues and macrophages of LPS-induced ALI mice, however, the mice lacking Bhlhe40 exhibited reduced lung tissue damage and inflammatory responses following LPS stimulation [49]. Additionally, The absence of Bhlhe40 suppressed GSDMD-driven pyroptosis and alleviated lung tissue damage by inhibiting caspase-11 noncanonical inflammasome-activated signaling pathways in LPS-induced ALI mice and macrophages [49]. These findings suggest that the functional collaboration of caspase-11 noncanonical inflammasome and Bhlhe40 is essential for LPS-induced ALI.

Wang et al. also demonstrated the pharmacological role of abscisic acid (ABA) in ARDS by targeting caspase-11 noncanonical inflammasome in human ARDS patients and LPS-induced ALI mice [50]. Plasma ABA levels were elevated in ARDS patients and LPS-induced ALI mice, and ABA reduced airway inflammation in ALI mice [50]. ABA suppressed the activation of the caspase-11 noncanonical inflammasome, thereby inhibiting the proteolytic activation of GSDMD and preventing GSDMD pore-mediated pyroptosis in alveolar macrophages within the lungs of ALI mice [50]. However, the protective effect of ABA on LPS-induced pyroptosis in alveolar macrophages was reversed by the overexpression of caspase-11 [50]. These results indicate that the caspase-11 noncanonical inflammasome plays a key role in ALI and ARDS by promoting pyroptosis in alveolar macrophages within lung tissues, a process mitigated by ABA.

The role of caspase-4/11 noncanonical inflammasomes in the development of ALI was further confirmed in LPS-challenged human lung endothelial cells and the endothelial cells from LPS-induced ALI mice. Cheng et al. explored the caspase-11 noncanonical inflammasome-mediated endothelial pyroptosis in LPS-induced ALI mice [51]. Systemic exposure to LPS triggered severe endothelial pyroptosis, which was mediated by the caspase-4 noncanonical inflammasome in human lung microvascular endothelial cells (hMVECs) and human pulmonary artery ECs (HPAECs) as well as by the caspase-11 noncanonical inflammasome in the mMVECs of the LPS-induced ALI mice [51]. In mice lacking caspase-11, bone marrow transplantation with wild-type hematopoietic cells did not prevent LPS-induced ALI [51], suggesting that nonhematopoietic caspase-11 noncanonical inflammasome plays a crucial role in LPS-induced ALI. Moreover, caspase-11-deficient endothelial cells reduced lung edema, neutrophil accumulation, and mortality caused by endotoxemia [51]. These findings indicate the essential role of endothelial pyroptosis in lung injury caused by endotoxemia and suggest that targeting noncanonical inflammasomes in endothelial cells could be a valuable therapeutic approach for ALI.

**Figure 5:**
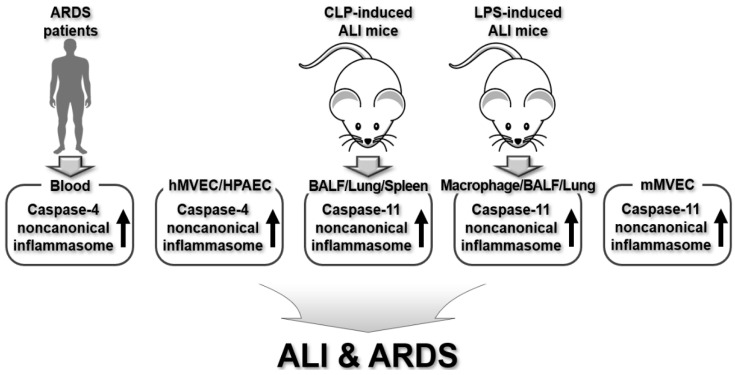
Regulatory roles of human caspase-4 and murine caspase-11 noncanonical inflammasomes in human ARDS patients, human lung microvascular endothelial cells, human pulmonary artery endothelial cells, and animal models of ALI.

In summary, caspase-4/11 noncanonical inflammasomes are crucial contributors to the development of ALI and ARDS, as evidenced by studies in human ARDS patients and mouse models of CLP- or LPS-induced ALI. Their regulatory roles in the pathogenesis of ALI and ARDS are summarized in Figure 5.

### 3.4. Idiopathic Pulmonary Fibrosis (IPF)

Idiopathic pulmonary fibrosis (IPF) is a chronic, progressive disease with substantial morbidity that affects the tissue surrounding the air sacs, or alveoli, in the lungs [52]. This condition develops when the lung tissues become thick and stiff for unknown reasons, and over time, these changes can cause permanent scarring in the lungs, called fibrosis, that makes it progressively more difficult to breathe [52]. For many years, IPF was thought to be a primarily inflammation-driven disease due to the observed increase in inflammatory cells within IPF lungs [52,53]. In a recent meta-analysis study, IPF affects about 3 million people worldwide, with a substantial increase in incidence with age [54]. There is no cure for IPF at present, and existing treatments only slow the disease’s progression, with a poor prognosis.

The role of noncanonical inflammasomes in the development of IPF has been shown in different lung epithelial cells and animal models that replicate human IPF. Terlizzi et al. reported the functional cooperation between AIM2 canonical and caspase-4 noncanonical inflammasomes in IPF pathogenesis using PBMCs from IPF patients and BLM-induced pulmonary fibrosis mice [55]. The pro-inflammatory cytokines IL-1β and IL-18 were elevated in PBMCs of IPF patients and mice with BLM-induced pulmonary fibrosis, with this increase linked to the release of the pro-fibrotic cytokine TGF-β through an AIM2 canonical inflammasome-dependent pathway [55]. Additionally, AIM2 activation triggered the release of caspase-4 from IPF-derived PBMCs, which corresponded to higher mRNA levels of this caspase in IPF PBMCs compared to healthy ones [55]. These findings revealed that the functional interaction between the AIM2 canonical and the caspase-4 noncanonical inflammasome drives pulmonary inflammation and fibrosis in both IPF patients and an animal model of the disease. Nevertheless, the mechanisms underlying the functional cooperation between these two inflammasomes remain unclear and require further investigation.

Peng et al. investigated the role of caspase-4/11 noncanonical inflammasomes in IPF development using lung epithelial cells and a mouse model of bleomycin (BLM)-induced pulmonary fibrosis. The levels of caspase-11, cleaved GSDMD, and IL-1β levels were significantly increased, which contributed to pulmonary inflammation in BLM-stimulated human and rat lung epithelial cells, as well as in BLM-induced pulmonary fibrosis mice [56]. Epithelial-mesenchymal transition (EMT) is a universal process in lung diseases with implications for fibrosis pathophysiology [57,58,59]. The levels of EMT-associated markers were elevated in BLM-stimulated lung epithelial cells and BLM-induced pulmonary fibrosis mice [56]. These results suggest that caspase-4/11 noncanonical inflammasomes contribute to the development of IPF by triggering pulmonary inflammation and fibrosis. However, the precise role and underlying mechanisms of noncanonical inflammasomes in IPF pathogenesis remain unclear and require further investigation.

Song et al. also investigated GSDMD-driven pyroptosis mediated by caspase-4/11 noncanonical inflammasomes in pulmonary inflammation and fibrosis, focusing on silicosis patients and silica-induced silicosis in mice [60]. GSDMD-mediated pyroptosis was observed in the lung tissues of both silicosis patients and mice [60]. Furthermore, silica exposure activated the caspase-11 noncanonical inflammasome, leading to IL-1β release and GSDMD-driven pyroptosis in silica-stimulated macrophages [60]. These findings indicate that macrophages underwent activation of caspase-4/11 noncanonical inflammasomes, resulting in GSDMD-dependent pyroptosis, which contributes to pulmonary inflammation and fibrosis during the progression of silicosis.

**Figure 6:**
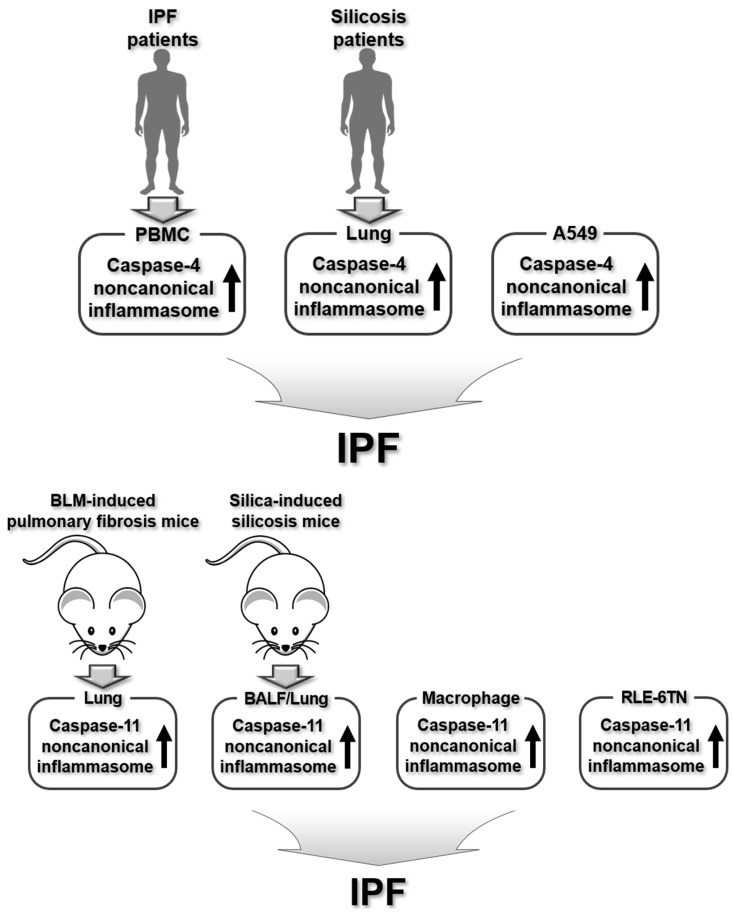
Regulatory roles of human caspase-4 and murine caspase-11 noncanonical inflammasomes in human IPF patients, human macrophages, animal models of pulmonary fibrosis, and murine macrophages and alveolar epithelial cells.

In summary, caspase-4/11 noncanonical inflammasomes play a key role in the pathogenesis of IPF pathogenesis, as shown in animal models of IPF and human IPF patients. The regulatory roles of caspase-4/11 noncanonical inflammasomes in IPF pathogenesis are summarized in Figure 6.

## 4. Conclusions and Perspectives

This review comprehensively discusses current research highlighting the regulatory functions of human caspase-4 and murine caspase-11 noncanonical inflammasomes in the development and progression of inflammatory lung diseases, including asthma, COPD, ALI/ARDS, and IPF, along with some of the underlying mechanisms using animal models of these diseases and human patients, as outlined in Table 1. While caspase-4/11 noncanonical inflammasomes have distinct roles associated with various molecules in each inflammatory lung disease, all the studies discussed in this review clearly demonstrate that these inflammasomes contribute to pulmonary inflammation, injury, and fibrosis by triggering inflammatory responses and GSDMD-mediated pyroptosis at disease lesions. This leads to the development and progression of inflammatory lung diseases, strongly indicating that targeting caspase-4/11 noncanonical inflammasomes could be a promising therapeutic strategy for these conditions.

While research has clearly highlighted the regulatory functions of noncanonical inflammasomes in inflammatory lung diseases, the majority of these studies have concentrated on the murine caspase-11 noncanonical inflammasome within mouse models of such diseases. This strongly indicates the need for further investigation into the regulatory role of the human caspase-4 noncanonical inflammasome in patients with various inflammatory lung diseases. Additionally, although there is compelling evidence that noncanonical inflammasomes play an active role in the development of inflammatory lung diseases, the molecular and cellular mechanisms driving their involvement, as well as their functional interactions with other molecules during disease progression, remain poorly understood. Consequently, future research should focus on identifying and validating cellular factors functionally associated with noncanonical inflammasomes and elucidating the mechanisms underlying their roles in the pathogenesis of these diseases. Moreover, there is a significant need for the development of potential therapeutics targeting noncanonical inflammasomes and for conducting translational studies to evaluate these treatments in human patients with inflammatory lung diseases. Finally, the regulatory roles of noncanonical inflammasomes in other lung diseases, including bronchitis, cystic fibrosis, pneumonia, tuberculosis, pulmonary edema, and lung cancers, warrant further investigation.

In conclusion, murine caspase-11 and human caspase-4 noncanonical inflammasomes play regulatory roles in the development and progression of various inflammatory lung diseases by interacting with other cellular factors. This regulation involves triggering inflammatory responses and tissue damage in disease sites through elevated pro-inflammatory cytokine levels and GSDMD-mediated pyroptosis. Gaining insight into the regulatory roles and underlying mechanisms of noncanonical inflammasomes in the pathogenesis of inflammatory lung diseases could contribute to the development of effective therapeutics targeting these inflammasomes and support clinical and translational research in patients with inflammatory lung diseases.

## Figures and Tables

**Table 1:** Regulatory roles of noncanonical inflammasomes in inflammatory lung diseases.

Diseases	Inflammasomes	Roles	Models	Ref.
Asthma	Caspase-11	HDM and allergy-associated cytokines increased the expression of caspase-11 in mouse BMDMs. Production of pro-inflammatory cytokines decreased in caspase-11^−/−^ BMDMs in response to HDM. Total cellular infiltration into BALF and pro-inflammatory cytokine levels in the BALF were significantly decreased in caspase-11^−/−^ mice in response to HDM. Histological signs of lung inflammation were reduced in caspase-11^−/−^ mice in response to HDM.	Mouse BMDMs HDM-induced allergic asthma mice	[32]
	Caspase-4	Neutrophilic asthma patients showed a marked increase in caspase-4 expression and elevated production of IL-1β and IL-18. Elevated expression and production of caspase-4, IL-1β, and IL-18 were not observed in other asthma types, such as eosinophilic and paucigranulocytic asthma.	Human neutrophilic asthma patients	[33]
	Caspase-4/11	Caspase-11 expression was upregulated in OVA-induced allergic lung inflammation in mice. Caspase-11 deficiency protected allergic lung inflammation in mice. PGE2 inhibited caspase-11 expression and activation of caspase-11 noncanonical inflammasome in mouse BMDMs. PGE2 inhibited caspase-11 and caspase-4 noncanonical inflammasome-driven pyroptosis in mouse BMDMs and human monocyte-derived macrophages.	Mouse BMDMs Human monocyte-derived macrophages OVA-induced allergic asthma mice Human asthma patients	[34]
		Dectin-1 activated noncanonical caspase-11 inflammasome and triggered pyroptosis, leading to pulmonary neutrophil inflammation in HDM-induced asthmatic mice and MH-S alveolar macrophages. Inhibition of caspase-11 noncanonical inflammasome alleviated Dectin-1-activated airway inflammation, proteolytic activation of GSDMD, and pyroptosis, leading to reduced neutrophil inflammation in HDM-induced asthmatic mice and MH-S alveolar macrophages. Dectin-1 expression in macrophages showed a positive correlation with neutrophil inflammation and caspase-4 expression in asthma patients.	Mouse MH-S cells HDM-induced allergic asthma mice Human asthma patients	[35]
COPD	Caspase-11	Deficiency of caspase-11 noncanonical inflammasome reduced pro-inflammatory cytokine levels in BALF and lung tissues of CS-challenged COPD mice. Deficiency of caspase-11 noncanonical inflammasome alleviated neutrophilia and airway inflammation in BALF and lung tissues of CS-challenged COPD mice.	CS-induced COPD mice	[37]
		Exposure to CS led to alveolar enlargement, collagen deposition, and an increase in mucus and pro-inflammatory cytokine production in mouse lung tissues. Caspase-11 noncanonical inflammasome was activated in lung tissues of CS-exposed mice. CS-induced alveolar enlargement, collagen deposition, and increase in mucus and pro-inflammatory cytokine production were reduced in lung tissues of 129Sv mice, which have nonfunctional caspase-11.	CS-induced COPD mice	[38]
	Caspase-4	The activation of the AIM2 inflammasome triggered the release of IL-1a and TGF-b in exacerbated PBMCs from COPD patients. The release of these two pro-inflammatory cytokines triggered by AIM2 inflammasome activation was dependent on a caspase-4 noncanonical inflammasome.	Human COPD patients	[39]
	Caspase-4/11	*M. catarrhalis* infection activated caspase-4/11 noncanonical inflammasomes in mouse BMDMs and THP-1 human macrophages. *M. catarrhalis* infection-induced GSDMD-dependent pyroptosis and NLRP3 canonical inflammasome activation in mouse BMDMs and THP-1 human macrophages	Mouse BMDMs Human THP-1 cells	[41]
ALI & ARDS	Caspase-11	HMGB1 aggravated lung injury and promoted severe inflammation in CLP-induced ALI mice. The expression of HMGB1 and caspase-11 increased in the lungs of CLP-induced ALI mice. Inhibition of HMGB1 decreased expression of caspase-11 and pyroptosis in the lungs of CLP-induced ALI mice.	CLP-induced ALI mice	[45]
		Lung injury and inflammation were diminished in ALI mice lacking HMGB1 and its receptor, RAGE. Lung injury and inflammation were also diminished in ALI mice lacking caspase-11.	CLP-induced ALI mice	[46]
		Luteolin reduced inflammation and lung injury in CLP-induced ALI mice. Levels of caspase-11, GSDMD, and pro-inflammatory cytokines were reduced in the lungs of CLP-induced ALI mice.	CLP-induced ALI mice	[47]
		Caspase-11 noncanonical inflammasome was activated, triggering inflammatory responses in the lungs of LPS-induced ALI mice and primary peritoneal macrophages. Caspase-11 noncanonical inflammasome activation was suppressed in LPS-induced ALI mice lacking CHOP. Caspase-11 noncanonical inflammasome activation was activated in response to an ER stress inducer.	Mouse peritoneal macrophages LPS-induced ALI mice	[48]
		Bhlhe40 was significantly expressed in lung tissues and macrophages of LPS-induced ALI mice. Mice lacking Bhlhe40 exhibited reduced lung tissue damage and inflammatory responses following LPS stimulation. Absence of Bhlhe40 suppressed GSDMD-driven pyroptosis and alleviated lung tissue damage by inhibiting caspase-11 noncanonical inflammasome-activated signaling pathways in LPS-induced ALI mice and macrophages.	Mouse BMDMs RAW264.7 cells LPS-induced ALI mice	[49]
		Plasma ABA levels were elevated in ARDS patients and LPS-induced ALI mice. ABA reduced airway inflammation in ALI mice. ABA suppressed caspase-11 noncanonical inflammasome activation, thereby inhibiting proteolytic activation of GSDMD and preventing GSDMD pore-mediated pyroptosis in alveolar macrophages within the lungs of ALI mice. Protective effect of ABA on LPS-induced pyroptosis in alveolar macrophages was reversed by the overexpression of caspase-11.	Mouse alveolar macrophages LPS-induced ALI mice Human ARDS patients	[50]
	Caspase-4/11	Systemic exposure to LPS triggered severe endothelial pyroptosis, which was mediated by the caspase-4 noncanonical inflammasome in human endothelial cells and the caspase-11 noncanonical inflammasome in LPS-induced ALI mice. In mice lacking caspase-11, bone marrow transplantation with wild-type hematopoietic cells did not prevent LPS-induced ALI. Caspase-11-deficient endothelial cells reduced lung edema, neutrophil accumulation, and mortality caused by LPS-mediated endotoxemia.	hMVECs HPAECs mMVECs LPS-induced ALI mice	[51]
IPF	Caspase-4	IL-1β and IL-18 levels were increased in PBMCs of IPF patients and mice with BLM-induced pulmonary fibrosis. The elevation of these pro-inflammatory cytokines was associated with the release of the pro-fibrotic cytokine TGF-β. AIM2 activation triggered the release of caspase-4 from IPF-derived PBMCs, which corresponded to higher mRNA levels of this caspase in IPF PBMCs compared to healthy ones.	BLM-induced pulmonary fibrosis mice Human IPF patients	[55]
	Caspase-4/11	Levels of caspase-11, cleaved GSDMD, and IL-1β levels were increased, which contributed to pulmonary inflammation in BLM-stimulated human and rat lung epithelial cells, as well as in BLM-induced pulmonary fibrosis mice. Levels of EMT-associated markers were elevated in BLM- stimulated lung epithelial cells and BLM-induced pulmonary fibrosis mice.	Human A549 cells Rat RLE-6TN cells BLM-induced pulmonary fibrosis mice	[56]
		GSDMD-mediated pyroptosis was observed in lung tissues of both silicosis patients and mice. Silica exposure activated the caspase-11 noncanonical inflammasome, leading to IL-1β release and GSDMD-driven pyroptosis in silica-stimulated macrophages.	Mouse peritoneal macrophages Silica-induced silicosis mice Human silicosis patients	[60]

## Data Availability

Not applicable.

## Abbreviations

COPD	Chronic obstructive pulmonary disease
ALI	Acute lung injury
ARDS	Acute respiratory distress syndrome
IPF	Idiopathic pulmonary fibrosis
PAMP	Pathogen-associated molecular pattern
DAMP	Damage-associated molecular pattern
PRR	Pattern-recognition receptor
GSDMD	Gasdermin D
HDM	House dust mites
BALF	Bronchial alveolar lavage fluid
CS	Cigarette smoke
CLP	Cecal ligation and puncture
LPS	Lipopolysaccharide
